# Effects of Various Pre-Treatment and Cooking on the Levels of Biogenic Amines in Korean and Norwegian Mackerel

**DOI:** 10.3390/foods10092190

**Published:** 2021-09-15

**Authors:** Yang-Su Kim, Yuri Kim, Hyunbeen Park, Jooyeon Park, Kwang-Geun Lee

**Affiliations:** 1 Department of Food Science and Biotechnology, Dongguk University-Seoul, 32, Dongguk-ro, Ilsandong-gu, Goyang-si 10326, Gyeonggi-do, Korea; high_code@naver.com (Y.-S.K.); yuri@kfri.re.kr (Y.K.); hbeen14@naver.com (H.P.); pjy041100@naver.com (J.P.); 2 Food Certification Support Centre, Korea Food Research Institute, 245, Nongsaengmyeong-ro, Iseo-myeon, Wanju-gun 55365, Jeollabuk-do, Korea

**Keywords:** mackerel, biogenic amine, high-performance liquid chromatography, pre-treatment, cooking

## Abstract

This study analyses the biogenic amines (BAs) formed in mackerel cooked by various methods and conditions. Five BAs, including tryptamine, β-phenylethylamine, putrescine, histamine, and spermidine, were analysed by high-performance liquid chromatography with UV detection. The level of total BAs was higher in the mackerel fillet (108.14 µg/g) than the headed and gutted fish (91.58 µg/g). Roasted, fried, and stewed mackerel recorded total BA concentrations of 54.28, 82.25, and 163.05 µg/g, respectively. Stewed mackerel contained about 3-fold more BAs than roasted mackerel. The level of total BAs in mackerel increased significantly up to 190%, 236% and 152% as the roasting temperature increased, upon frying, and as stewing temperature increased, respectively (*p* < 0.05).

## 1. Introduction

Mackerel is one of the most popular fish consumed in Korea. Safety issues for fishery products are increasing every year. Safety investigations on radioactive, heavy metal, and pesticide residues have been conducted for fishery products [1,2]. However, no study has yet conducted a detailed determination of biogenic amines (BAs) in cooked foods such as, for example, school meals. BAs are nitrogenous, low-molecular-weight organic bases with aromatic or heterocyclic structures. BAs are natural contaminants derived from the enzymatic decarboxylation of the amino acids concerned [3,4]. BAs can be formed in a variety of foods, such as cheese, sausage, fishery products, soybean products, and other fermented foods [5].

The BAs that affect the human body are tryptamine (TRP), histamine (HIS), putrescine (PUT), spermidine (SPD), and β-phenylethylamine (PHE), which are derived from amino acid precursors [5]. High levels of BAs can damage the human nervous and cardiovascular systems. BAs are known as potential precursors to carcinogenic nitrosamines [6,7,8]. Scombroid fish are characterised by the presence of histidine, a precursor of HIS in muscle tissue, more than other fish. Consuming food containing large amounts of HIS causes HIS poisoning, also called scombroid poisoning [9,10].

BAs are generally known to be heat-stable compounds. However, this is still controversial. Recent studies have indicated that the corresponding amino acid precursors can be formed by oxidative decarboxylation during the heating process [11]. Studies have reported reduced or increased BAs depending on the heat treatment temperature and time [12,13].

There are several studies investigating concentrations of biogenic amines in raw mackerel [14,15,16,17]. Recent studies reported that concentrations of biogenic amines in Indian mackerel of the tropical region during storage at ambient (25–29 °C) and ice temperature (0 °C) are related with changes of amino acids content and amines forming bacteria [18]. Although most people consume cooked mackerel, the contents of BAs from cooked mackerel have not been fully explored. Therefore, this study would be meaningful by analyzing the biogenic amine content of mackerel cooked in three different ways (roasting, frying, stewing).

In this study, to assess how various conditions can affect BAs in chub mackerel (*Scomber japonicus*), five BAs, including TRP, PHE, PUT, HIS, and SPD, formed in mackerel were analyzed considering its origin (Korea and Norway), pre-treatment (headed and gutted [H/G], fillet) and cooking method (roasted, fried, stewed).

## 2. Materials and Methods

### 2.1. Chemicals and Materials

Mackerel (*S. japonicus* from Korea and Norway) was purchased from Namyang Seafood Co. (Sungnam, Korea). Mackerel was purchased within a year of being caught and was kept at −18 °C until sample preparation. Canned tuna (Dongwon Industries Co., Seoul, Korea) was purchased in the general market in Seoul, Korea, and used to validate the five BAs.

Putrescine dihydrochloride, histamine dihydrochloride, TRP, 2-phenylethylamine, spermidine tri-hydrochloride, 1,7-diaminoheptane, perchloric acid, sodium hydrogen carbonate, and ammonium hydroxide were purchased from Sigma Aldrich Chemical Co. (St. Louis, MO, USA). HPLC-grade water and acetonitrile were purchased from J.T. Baker (Phillips, NJ, USA). The dansyl chloride reagent used for derivatisation was purchased from Tokyo Chemical Industry Co., Ltd. (Tokyo, Japan).

### 2.2. Validation of the Analysis of BAs

Canned tuna was used to validate the five BAs since it has a similar fatty acid composition to mackerel and has not been reported to have BAs. For method validation, linearity (coefficient of determination, R^2^ of the calibration curve), limit of detection (LOD), limit of quantitation (LOQ), recovery (%), and precision (relative standard deviation, RSD %) were determined. The calibration curve of the five BAs in canned tuna was prepared by measuring seven concentrations of the BAs standard solution (0, 1, 3, 5, 10, 50, 100, 300 µg/g). LOD and LOQ were calculated by the respective equations, 3.14 × standard deviation (σ1)/slope factor and 10 × standard deviation (σ1)/slope factor of the BAs calibration curve derived from eight points (0, 1, 3, 5, 10, 50, 100, 300 µg/g). For accuracy (%) and precision, the level of BAs were determined in canned tuna samples without internal standards. The intra-day accuracy and precision were analysed on one day by performing five replicates at each level of the BAs (10, 50, and 100 µg/g). The inter-day accuracy and precision were tested once a day for 5 days at each level of the BAs (10, 50, and 100 µg/g).

### 2.3. Preparation of Mackerel Samples

A total of 40 samples of mackerel, as shown in Table 1, were prepared for BAs analysis by origin (Korea and Norway), pre-treatment (H/G, fillet), cooking method (roasted, fried, stewed), temperature, and time conditions. Each treatment has 3 replications, and the total analytical samples were 120.

Frozen whole mackerel was thawed in water for 30 min before the experiments. H/G mackerel has viscera (guts) and head removed. Mackerel fillets were cut along the entire side of the fish, removing the spine and most bones from the meat. The cooking method applied to mackerel was conducted with reference to the Korean Dietetic Association (The Korean Dietary Association, 2007). Roasted mackerel was prepared by roasting 50 g of mackerel at 150, 200, and 250 °C for 15 min in an oven (HSB-N361B, Samsung, Seoul, Korea). For fried mackerel, 50 g of mackerel was fried in soybean oil (Sajo Haepyo, Seoul, Korea) at 140, 170, and 200 °C for 10 min using a fryer (DK-260, Delki, Seoul, Korea). For stewed mackerel, 50 g of mackerel, 200 mL of soy sauce (Sampyo, Seoul, Korea), and 200 mL of water were combined and boiled at 75, 85, and 95 °C for 15 min. A calibrated infrared thermometer (SATO-8700, SATO, Seoul, Korea) was used for temperature measurement in all cooking procedures. Prepared samples were stored at 4 °C for 3 h until analysis. The sample preparation of mackerel is described in Table 1.

### 2.4. Sample Preparation for BAs Analysis

#### 2.4.1. Preparation of Sample Extracts

The BAs analysis of mackerel was carried out according to the procedure proposed by Lee et al. [19]. In a 50 mL conical tube, 5 mL of mackerel sample was added to 20 mL of 0.4 M perchloric acid, and 50 μL of 1,7-diaminoheptane (10 g/L) as the internal standard. This mixture was homogenised using a vortex mixer, then left to react in a cold chamber for 2 h, followed by centrifugation at 12,000 rpm, 4 °C, for 10 min. The supernatant was collected, and the residue was re-extracted with 0.4 M perchloric acid of the same volume as the previous experimental method (second extraction). After pooling the two supernatants, the final volume was adjusted to 50 mL with 0.4 M perchloric acid and filtered through Whatman filter paper No. 1 (Whatman Ltd., Little Chalfont, UK) to progress the derivatisation.

#### 2.4.2. Derivatisation of Extracted Sample

BAs were derivatised according to the previously published method [20]. One millilitre of the extracted sample was mixed with 200 µL of 2 M NaOH and 300 µL of saturated sodium bicarbonate. For derivatisation, 2 mL of dansyl chloride solution was added to the mixture, followed by incubation of the mixture at 40 °C for 45 min. Ammonium hydroxide (25%, 100 µL) was added to stop the reaction and remove the residual dansyl chloride. After that, the mixture was stored in the dark at room temperature for 30 min, and then acetonitrile was added to adjust the final volume to 5 mL. The mixture was centrifuged at 3500 rpm for 5 min. The resultant supernatant was filtered through a syringe filter (25 mm, 0.2-μm pore size; Whatman Ltd.) and stored at −25 °C until analysis by HPLC-UV.

### 2.5. Analysis of BAs in Mackerel by HPLC-UV

The BAs were quantitated using an HPLC 1200 Series (Agilent Technologies, Santa Clara, CA, USA) equipped with a UV-Vis detector and Nova-Pack C18 column (3.9 × 150 mm, 4 µm). The mobile phases were 0.1 M ammonium acetate (solvent A) and acetonitrile (solvent B) at the flow rate of 1 mL/min with gradient elution for 25 min. The injection volume was 20 μL. The sample was detected by UV at a wavelength of 254 nm. After 25 min, the gradient was re-adjusted to 50% solvent A and 50% solvent B.

### 2.6. Statistical Analysis

The results were expressed as mean ± standard deviation of three measurements. Triplicate analyses were performed for each mackerel sample. Data were evaluated for origin, cooking methods, and temperature by one-way analysis of variance (ANOVA) and Duncan’s multiple range test using IBM SPSS Statistics 23 (IBM Co., Armonk, NY, USA). We ran a One-way ANOVA followed by Duncan’s multiple range test because we were interested in the effect of each independent variable (origin, cooking method, temperature) rather than the interaction between them. While it seems like temperature is nested in the cooking method, each variable was specifically selected for each cooking method; therefore, cooking method and temperature need to be treated independently. 

## 3. Results and Discussion

### 3.1. Validation of Analytical Method for BAs

For method validation, the results of linearity (coefficient of determination, R^2^ of the calibration curve), limit of detection (LOD), limit of quantitation (LOQ), recovery (%), and precision (relative standard deviation, RSD %) can be found in Table 2. Suitable linearity for BAs was observed at all concentrations. (R^2^ > 0.99) The measured LOD and LOQ ranges of the five BAs were 0.78–1.45 and 1.65–4.40 µg/g, respectively. Recovery (%) and precision (%) were measured, respectively, using three concentrations (10, 50, 100 µg/g) of the BAs standard solutions. The recovery rate ranged from 85.78% to 118.06%, the inter-day and intra-day precision values ranged from 0.95% to 9.68% and from 0.82% to 8.65%, respectively. The validation data of this study was similar to the previous reports [7,19].

### 3.2. Analysis of BAs Level in Mackerel

The concentrations of the five BAs detected in the 40 mackerel samples are presented in Table 3, Table 4 and Table 5. Though the stored frozen time of the fish before the experiment could have affected the experiment, the effect is not expected to confound the results since it applies to both origins and across treatments. Based on the cooking method, the concentration of the total BAs was highest in stewed (163.05 µg/g), followed by fried (82.25 µg/g) and roasted (54.28 µg/g). The highest concentration of total BAs (192.53 µg/g) was measured in the fillet of mackerel from Norway, stewed at 95 °C. The lowest total concentration (33.09 µg/g) was measured in the H/G fish from Korea, fried at 140 °C.

In the 40 mackerel samples, the concentration ranges of PUT, HIS, TRP, PHE, and SPD were 11.43–43.11, 6.34–84.2, 4.12–45.51, 4.01–42.87, and 2.13–10.09 µg/g, respectively. Among the cooking methods, the level of BAs was highest in stewed mackerel samples (*n* = 12) with 126.84–192.53 µg/g. Roasted mackerel had 34.6–93.32 µg/g (*n* = 12), and fried mackerel (*n* = 12) recorded 33.09–100.28 µg/g. By origin, the total BAs content was higher in pre-treated mackerel from Norway (51.81 ± 1.80µg/g) than Korea (45.09 ± 1.81 µg/g). The BA levels of fillet and H/G fish from Korea were lower than Norway by 14% and 15%, respectively.

In pre-treated mackerel, the content of total BAs was higher in the fillet (51.01 ± 0.94 µg/g) than the H/G fish (46.9 ± 2.38 µg/g). In the previous study dealing with biogenic amines formation and its relation to microbiological and sensory attributes in ice-stored whole, gutted, and filleted Mediterranean Sea bass, the number of microorganisms in fillet was more than headed and gutted part [21]. BAs are formed through bacterial enzyme activity. Fresh fish have low levels of BAs, with BA accumulation related to spoilage. The type and level of BAs in fish depend on the extent of spoilage, the specific spoilage organisms, and their counts. The increase in microbial counts due to post-processing contamination and storage time has led to a substantial rise in certain BAs [21].

The reason that stewed mackerel recorded the highest level of BAs (163.05 µg/g) by the cooking method can be explained by the presence of BAs in the soy sauce or soybean paste [22,23]. According to Yoon et al., the concentration of BAs was increased in Korean fermented foods [23]. In fermented products, such as soy sauce, soybean paste, and cheese, BAs are formed by the fermentation or decay of foods with a high protein content [19].

In all cooking methods, the level of total BAs increased significantly as the temperature increased. Increases of up to 190%, 236%, and 152% were observed as roasting, frying and stewing temperature increased, respectively (*p* < 0.05 for all). Since there has not been reports about the effect of cooking on the BA formation in fishery products it was hard to evaluate our results. However, in the roasting of cocoa beans, the highest temperature, and air humidity led to the greatest rise in BA due to precursor transformation at high heat treatment [13]. Referring to the study, it is speculated that steamed mackerel had the highest BA content because it had the highest humidity of the three cooking methods. In the study of soybean paste, roasting is attributed to increasing BA [8].

Additionally, recent studies have shown that BA can be formed during heat treatment by chemical decarboxylation of amino acids in the presence of lipid peroxides [24]. Therefore, the frying method seems to have a higher BA content than the roasting method due to the lipid peroxides.

In this study, five BAs, including TRP, PHE, PUT, HIS, and SPD, formed in mackerel subjected to various cooking methods and conditions were analysed. In 40 mackerel samples, the detected concentration ranges of PUT, HIS, TRP, PHE, and SPD were 11.43–43.11, 6.34–84.2, 4.12–45.51, 4.01–42.87, and 2.13–10.09 µg/g, respectively. The total BAs formed during roasting, frying, and stewing were 42.01–93.32, 33.09–146.58, and 126.84–19.53 µg/g, respectively. The total level of BAs increased significantly with increasing temperature in all cooking methods. Compared to the control group, the total BAs for roasting, frying, and stewing increased up to 170%, 311%, and 398%, respectively, when cooking was completed.

The results of this study indicate that a change of the cooking conditions such as temperature and time may contribute to the reduction of BA concentration in foods. Future studies should include a comprehensive analysis of the BA levels in foods using various western and eastern cooking recipes for controlling the formation and dietary exposure to BAs.

## Figures and Tables

**Table 1:** Preparation of 40 mackerel samples.

Origin	Pre-Treatment	Cooking Method	Temperature	Time
Korea	Headed and gutted (H/G)	Unhandled (control)		
		Roasting	150 °C	15 min
			200 °C	
			250 °C	
		Frying	140 °C	10 min
			170 °C	
			200 °C	
		Stewing	95 °C	15 min
			85 °C	
			75 °C	
	Fillet	Unhandled (control)		
		Roasting	150 °C	15 min
			200 °C	
			250 °C	
		Frying	140 °C	10 min
			170 °C	
			200 °C	
		Stewing	95 °C	15 min
			85 °C	
			75 °C	
Norway	Headed and gutted (H/G)	Unhandled (control)		
		Roasting	150 °C	15 min
			200 °C	
			250 °C	
		Frying	140 °C	10 min
			170 °C	
			200 °C	
		Stewing	95 °C	15 min
			85 °C	
			75 °C	
	Fillet	Unhandled (control)		
		Roasting	150 °C	15 min
			200 °C	
			250 °C	
		Frying	140 °C	10 min
			170 °C	
			200 °C	
		Stewing	95 °C	15 min
			85 °C	
			75 °C	

**Table 2:** Validation for the analysis of BAs (calibration curve, linearity, LOD, LOQ, RSD (%), recovery rate).

BAs ^b^	Calibration Curve ^a^		LOD ^c^ (µg/g)	LOQ ^d^ (µg/g)	Precision (RSD %)						Recovery Rate (%)		
					Inter-Day			Intra-Day					
	Equation (y = ax + b)	Linearity (R^2^)			Concentration (µg/g)			Concentration (µg/g)			Concentration (µg/g)		
					10	50	100	10	50	100	10	50	100
TRP	y = 0.0123x + 0.0493	0.9996	0.78	2.49	3.46	7.64	4.66	8.65	6.34	3.53	98.23	106.38	89.62
PHE	y = 0.0113x + 0.0464	0.9997	0.52	1.65	9.68	3.73	0.95	1.54	4.81	2.86	85.78	107.62	95.87
PUT	y = 0.0046x + 0.088	0.9985	0.84	2.69	4.89	2.26	1.84	2.76	0.82	2.94	113.25	118.06	102.85
HIS	y = 0.0039x + 0.0165	0.9999	0.65	2.08	2.74	1.08	2.62	1.88	3.46	4.85	94.85	97.68	93.85
SPD	y = 0.0009x + 0.0065	0.9991	1.45	4.4	5.85	1.87	3.87	6.74	3.08	5.64	103.51	102.68	111.93

^a^ Range of calibration curve was measured at seven points: 0.1, 3.5, 10, 50, 100, 300 (µg/g). ^b^ Trp: tryptamine, Phe: β-phenylethylamine, Put: putrescine, His: histamine, Spd: spermidine. ^c^ LOD = 3.14 × Standard deviation/Slope (µg/g). ^d^ LOQ = 10 × Standard deviation/Slope (µg/g).

**Table 3:** Concentrations of BAs in roasted mackerel samples.

Origin	Pretreatment & Cooking Method	Temperature	Tryptamine (TRP)	β-Phenyl Ethylamine (PHE)	Putrescine (PUT)	Histamine (HIS)	Spermidine (SPD)	Total BAs
Korea	Control(H/G)		8.03 ± 0.95	4.01 ± 0.41	22.32 ± 2.1	6.34 ± 0.17	2.30 ± 0.22	43.00 ± 3.24
	Control(fillet)		8.3 ± 0.77	5.27 ± 0.32	21.62 ± 2.15	8.38 ± 0.05	3.6 ± 0.11	47.18 ± 1.63
	Headed and gutted, Roasted	150 °C	5.10 ± 0.09 ^a,A,α^	7.07 ± 0.25 ^b,B,βγ^	20.95 ± 2.8 ^a,B α^	6.45 ± 0.26 ^a,A,α^	2.44 ± 0.13 ^ab,A,α^	42.01 ± 3.27
		200 °C	6.74 ± 0.35 ^a,A,βγ^	6.53 ± 0.24 ^a,A,α^	26.23 ± 3.32 ^a,B,β^	7.01 ± 0.26 ^ab,A,αβ^	2.86 ± 0.06 ^a,A,αβ^	49.37 ± 4.06
		250 °C	6.07 ± 0.5 ^a,A,β^	6.41 ± 0.62 ^a,A,αβ^	30.40 ± 0.91 ^b,AB,γ^	7.49 ± 0.16 ^ab,A,βγ^	3.31 ± 0.13 ^a,A,βγ^	53.68 ± 1.07
	Fillet, Roasted	150 °C	5.20 ± 0.39 ^a,B,α^	5.95 ± 0.7 ^a,A,α^	11.43 ± 0.6 ^a,A,α^	9.04 ± 0.65 ^b,B,α^	2.98 ± 0.61 ^b,B,α^	34.60 ± 1.65
		200 °C	14.08 ± 0.79 ^a,B,αβ^	6.86 ± 0.39 ^b,B,αβ^	13.05 ± 0.53 ^ab,A,αβ^	11.13 ± 0.24 ^a,B,αβ^	3.77 ± 0.34 ^b,B,β^	48.89 ± 1.17
		250 °C	22.29 ± 1.57 ^a,B,βγ^	7.99 ± 0.53 ^a,B,βγ^	14.91 ± 0.76 ^a,A,β^	13.84 ± 1.06 ^ab,B,γ^	3.88 ± 0.37 ^b,B,βγ^	62.91 ± 2.35
Norway	Control(H/G)		6.46 ± 0.6	4.17 ± 0.09	28.08 ± 3.23	6.49 ± 0.05	5.61 ± 0.5	50.80 ± 3.49
	Control(fillet)		9.85 ± 0.81	5.66 ± 0.41	23.74 ± 0.26	8.82 ± 0.07	6.77 ± 0.28	54.83 ± 0.94
	Headed and gutted, Roasted	150 °C	5.96 ± 0.5 ^b,A,α^	5.36 ± 0.1 ^a,A,α^	22.10 ± 1.24 ^ab,B,α^	6.35 ± 0.23 ^a,A,α^	2.28 ± 0.06 ^a,AB,α^	42.06 ± 1.8
		200 °C	7.06 ± 0.36 ^a,A,αβ^	8.56 ± 0.38 ^b,B,βγ^	27.93 ± 3.34 ^b,B,β^	6.66 ± 0.53 ^a,A,α^	4.04 ± 0.14 ^b,B,βγ^	54.25 ± 2.55
		250 °C	7.35 ± 0.84 ^b,A,αβ^	7.62 ± 1.07 ^b,A,β^	29.10 ± 0.57 ^a,b,γ^	7.06 ± 0.07 ^a,A,αβ^	4.35 ± 0.34 ^b,B,γ^	55.48 ± 2.27
	Fillet, Roasted	150 °C	18.28 ± 1.41 ^b,B,α^	7.19 ± 0.3 ^b,B,α^	13.24 ± 0.29 ^ab,A,αβ^	8.32 ± 0.68 ^a,B,α^	2.13 ± 0.07 ^a,A,α^	49.17 ± 0.66
		200 °C	28.10 ± 2.99 ^b,B,β^	7.80 ± 0.25 ^a,A,αβ^	15.53 ± 0.88 ^b,A,β^	12.04 ± 0.73 ^b,B,β^	2.15 ± 0.07 ^a,A,α^	65.62 ± 3.44
		250 °C	31.11 ± 2.04 ^b,B,β^	10.16 ± 0.75 ^b,B,γ^	34.08 ± 2.68 ^b,B,γ^	14.42 ± 0.91 ^b,B,γ^	3.55 ± 0.15 ^a,A,β^	93.32 ± 1.56

Mean Values with three types of superscripts: ^a,b^—indicate significant differences in origin; ^A,B^—indicate significant differences in pre-treatment; ^α,β,γ^ – indicate significant differences in Temperature. The significant differences according to Duncan’s test (*p* < 0.05).

**Table 4:** Concentrations of BAs in fried mackerel samples.

Origin	Pretreatment & Cooking Method	Temperature	Tryptamine (TRP)	β-Phenyl Ethylamine (PHE)	Putrescine (PUT)	Histamine (HIS)	Spermidine (SPD)	Total BAs
Korea	Control(H/G)		8.03 ± 0.95	4.01 ± 0.41	22.32 ± 2.1	6.34 ± 0.17	2.30 ± 0.22	43.00 ± 3.24
	Control(fillet)		8.3 ± 0.77	5.27 ± 0.32	21.62 ± 2.15	8.38 ± 0.05	3.6 ± 0.11	47.18 ± 1.63
	Headed and gutted, Fried	140 °C	4.12 ± 0.42 ^a,A,α^	5.92 ± 0.29 ^a,A,α^	12.22 ± 0.91 ^a,A,α^	8.32 ± 0.95	2.51 ± 0.53 ^a,A,α^	33.09 ± 2.84
		170 °C	12.88 ± 0.48 ^a,A,β^	11.08 ± 0.75 ^b,A,β^	16.45 ± 1.92 ^a,A,β^	10.78 ± 0.69 ^a,A, β^	3.71 ± 0.3 ^a,A,β^	54.89 ± 3.49
		200 °C	17.70 ± 2.3 ^a,A,γ^	20.83 ± 0.69 ^a,A,γ^	20.00 ± 2.27 ^b,A,γ^	13.86 ± 0.88 ^a,A, γ^	4.90 ± 0.62 ^a,A,γ^	77.28 ± 5.35
	Fillet, Fried	140 °C	31.30 ± 3.27 ^b,B,β^	19.20 ± 2.28 ^b,B,β^	34.02 ± 4.25 ^b,B,α^	15.16 ± 1.53 ^a,B, α^	10.09 ± 0.77 ^b,B,γ^	109.77 ± 2.43
		170 °C	29.96 ± 1.99 ^b,B,α^	16.96 ± 0.74 ^a,B,α^	22.91 ± 1.14 ^b,B,β^	22.70 ± 0.16 ^a,A,α^	9.30 ± 0.69 ^b,B,β^	101.84 ± 3.93
		200 °C	45.51 ± 5.59 ^a,AB,γ^	22.39 ± 1.97 ^b,B,γ^	31.25 ± 1.06 ^b,B,β^	40.20 ± 3.8 ^b,B,β^	7.22 ± 0.18 ^b,B,α^	146.58 ± 6.43
Norway	Control(H/G)		6.46 ± 0.6	4.17 ± 0.09	28.08 ± 3.23	6.49 ± 0.05	5.61 ± 0.5	50.80 ± 3.49
	Control(fillet)		9.85 ± 0.81	5.66 ± 0.41	23.74 ± 0.26	8.82 ± 0.07	6.77 ± 0.28	54.83 ± 0.94
	Headed and gutted, Fried	140 °C	9.33 ± 0.4 ^b,A,α^	7.79 ± 0.77 ^a,A,α^	13.49 ± 1.26 ^b,A,α^	8.54 ± 0.37 ^b,A,α^	2.87 ± 0.27 ^b,A,α^	42.03 ± 1.33
		170 °C	19.89 ± 2.2 ^b,A,β^	10.21 ± 0.79 ^a,A,β^	17.37 ± 1.63 ^b,A,β^	11.52 ± 0.75 ^a,A,β^	8.29 ± 0.88 ^a,B,β^	67.28 ± 5.55
		200 °C	24.32 ± 2.03 ^b,A,γ^	32.27 ± 3.04 ^b,B,γ^	18.52 ± 1.68 ^a,A,γ^	15.30 ± 1.94 ^b,A,γ^	8.91 ± 0.77 ^b,B,γ^	99.32 ± 4.89
	Fillet, Fried	140 °C	16.82 ± 2.19 ^a,B,α^	15.70 ± 1.66 ^a,B,β^	20.48 ± 1.08 ^a,B,β^	10.29 ± 0.37 ^a,B,α^	6.04 ± 0.06 ^a,B,γ^	69.33 ± 5.12
		170 °C	24.19 ± 3.03 ^a,B,β^	21.55 ± 2.07 ^b,B,γ^	18.82 ± 2.15 ^a,B,α^	14.83 ± 1.12 ^b,B,β^	5.93 ± 0.22 ^b,A,β^	85.31 ± 3.83
		200 °C	37.63 ± 5.02 ^a,B,γ^	14.97 ± 1.64 ^a,A,α^	21.19 ± 1.96 ^a,B,β^	24.32 ± 2.05 ^a,B,γ^	2.16 ± 0.11 ^a,A,α^	100.28 ± 7.4

Mean Values with three types of superscripts: ^a,b^—indicate significant differences in origin; ^A,B^—indicate significant differences in pre-treatment; ^α,β,γ^ —indicate significant differences in Temperature. The significant differences according to Duncan’s test (*p* <0.05).

**Table 5:** Concentrations of BAs in stewed mackerel samples.

Origin	Pretreatment & Cooking Method	Temperature	Tryptamine (TRP)	β-Phenyl Ethylamine (PHE)	Putrescine (PUT)	Histamine (HIS)	Spermidine (SPD)	Total BAs
Korea	Control(H/G)		8.03 ± 0.95	4.01 ± 0.41	22.32 ± 2.1	6.34 ± 0.17	2.30 ± 0.22	43.00 ± 3.24
	Control(fillet)		8.3 ± 0.77	5.27 ± 0.32	21.62 ± 2.15	8.38 ± 0.05	3.6 ± 0.11	47.18 ± 1.63
	Headed and gutted, Stewed	75 °C	17.41 ± 1.06 ^a,A,α^	24.62 ± 4.35 ^a,AB,α^	43.11 ± 4.29 ^b,B,α^	58.18 ± 6.14 ^b,B,α^	8.39 ± 0.83 ^b,B,αβ^	151.70 ± 4.35
		85 °C	22.21 ± 1.39 ^b,B,β^	36.88 ± 2.83 ^a,A,α^	42.39 ± 3.14 ^a,A,β^	64.81 ± 2.28 ^a,A,αβ^	8.66 ± 0.21 ^b,AB,βγ^	174.94 ± 9.18
		95 °C	21.41 ± 0.15 ^a,B,βγ^	39.2 ± 2.63 ^a,A,β^	43.09 ± 1.15 ^ab,B,γ^	73.65 ± 3.21 ^ab,A,γ^	9.85 ± 0.21 ^b,A,γ^	187.21 ± 3.51
	Fillet, Stewed	75 °C	23.7 ± 2.2 ^a,A,αβ^	32.6 ± 1.57 ^b,A,β^	39.89 ± 4.15 ^a,A,α^	53.5 ± 6.48 ^b,A,α^	7.73 ± 0.61 ^a,A,α^	157.43 ± 12.06
		85 °C	21.59 ± 1.23 ^b,A,βγ^	35.92 ± 2.33 ^b,A,αβ^	41.39 ± 2.35 ^b,B,β,γ^	66.87 ± 7.56 ^b,A,β^	8.46 ± 0.13 ^b,A,βγ^	174.23 ± 8.87
		95 °C	20.77 ± 0.26 ^a,A,αβγ^	42.87 ± 2.30 ^a,B,β^	42.89 ± 2.46 ^a,B,αβ^	71.61 ± 3.50 ^ab,AB,γ^	9.59 ± 0.22 ^b,A,γ^	187.73 ± 2.95
Norway	Control(H/G)		6.46 ± 0.6	4.17 ± 0.09	28.08 ± 3.23	6.49 ± 0.05	5.61 ± 0.5	50.80 ± 3.49
	Control(fillet)		9.85 ± 0.81	5.66 ± 0.41	23.74 ± 0.26	8.82 ± 0.07	6.77 ± 0.28	54.83 ± 0.94
	Headed and gutted, Stewed	75 °C	18.06 ± 1.12 ^a,AB,αβ^	29.97 ± 0.99 ^a,A,β^	39.91 ± 3.51 ^a,A,β^	43.72 ± 2.67 ^b,B,α^	5.86 ± 0.06 ^a,A,α^	137.52 ± 4.34
		85 °C	16.89 ± 0.91 ^a,A,α^	28.88 ± 1.57 ^a,A,αβ^	41.95 ± 4.12 ^b,B,β^	48.39 ± 3.03 ^a,A,αβ^	6.06 ± 0.55 ^a,A,αβ^	142.17 ± 2.26
		95 °C	22.69 ± 1.25 ^b,A,γ^	37.77 ± 1.68 ^b,B,β^	40.03 ± 0.52 ^a,B,α^	76.95 ± 6.69 ^a,AB,γ^	6.6 ± 0.15 ^b,A,γ^	184.04 ± 3.33
	Fillet, Stewed	75 °C	18.03 ± 0.65 ^a,A,αβ^	28.21 ± 2.70 ^a,A,α^	35.84 ± 2.17 ^b,A,γ^	39.11 ± 0.84 ^a,A,α^	5.65 ± 0.27 ^a,AB,αβ^	126.84 ± 3.06
		85 °C	17.11 ± 0.71 ^a,AB,α^	29.24 ± 1.67 ^a,A,β^	42.41 ± 3.58 ^b,A,β^	45.31 ± 1.72 ^a,A,αβ^	6.13 ± 0.51 ^a,A,α^	140.2 ± 1.29
		95 °C	22.86 ± 0.66 ^b,A,γ^	38.53 ± 0.74 ^b,A,βγ^	40.3 ± 0.45 ^a,A,β^	84.2 ± 2.53 ^a,A,γ^	6.64 ± 0.04 ^a,A,αβ^	192.53 ± 2.90

Mean Values with three types of superscripts: ^a,b^—indicate significant differences in origin; ^A,B^—indicate significant differences in pre-treatment; ^α,β,γ^—indicate significant differences in Temperature. The significant differences according to Duncan’s test (*p* < 0.05).
